# Global epidemiology of azole resistance in *Aspergillus fumigatus*

**DOI:** 10.1093/jacamr/dlaf219

**Published:** 2026-02-12

**Authors:** Anneke Fengler, Oliver Bader, Oliver Cornely, Oliver Kurzai, Jacques F Meis, Florent Morio, Axel Hamprecht

**Affiliations:** Institute of Medical Microbiology and Virology, Carl von Ossietzky Universität Oldenburg, Oldenburg, Germany; Institute for Medical Microbiology and Virology, University Medical Center Göttingen, Göttingen, Germany; Institute of Translational Research, Cologne Excellence Cluster on Cellular Stress Responses in Aging-Associated Diseases (CECAD), Faculty of Medicine, University of Cologne, Cologne, Germany; Department of Internal Medicine, Division of Infectious Diseases, Excellence Center for Medical Mycology (ECMM), University Hospital Cologne, Cologne, Germany; German Centre for Infection Research (DZIF), Partner Site Bonn-Cologne, Cologne, Germany; Institute for Hygiene and Microbiology, University of Würzburg, Würzburg, Germany; National Reference Center for Invasive Fungal Infections NRZMyk, Leibniz Institute for Natural Product Research and Infection Biology—Hans-Knoell-Institute, Jena, Germany; Institute of Translational Research, Cologne Excellence Cluster on Cellular Stress Responses in Aging-Associated Diseases (CECAD), Faculty of Medicine, University of Cologne, Cologne, Germany; Department of Internal Medicine, Division of Infectious Diseases, Excellence Center for Medical Mycology (ECMM), University Hospital Cologne, Cologne, Germany; German Centre for Infection Research (DZIF), Partner Site Bonn-Cologne, Cologne, Germany; Department of Medical Microbiology, Radboudumc and Centre for Expertise in Mycology, Radboudumc/CWZ, Nijmegen, The Netherlands; Département de mycologie médicale, Nantes Université, CHU Nantes, Cibles et Médicaments des Infections et de L'Immunité, IICiMed, UR 1155, Nantes 44000, France; Institute of Medical Microbiology and Virology, Carl von Ossietzky Universität Oldenburg, Oldenburg, Germany

## Abstract

The fungus *Aspergillus fumigatus* has evolved as an important cause of opportunistic fungal diseases in humans worldwide. It is the most frequent filamentous fungus colonizing the airways of patients with cystic fibrosis and can affect immunocompetent as well as immunocompromised individuals. *Aspergillus* disease is commonly treated with azoles, which inhibit lanosterol 14α-demethylase, a key component of the ergosterol biosynthesis pathway, essential for membrane integrity and fluidity. Lanosterol 14α-demethylase is encoded by the *CYP51A* gene. Azole-resistant *A. fumigatus* isolates often show amino acid substitutions in Cyp51A. Most prevalent mutations have a tandem repeat (TR) in the promoter and point mutations in the gene (e.g. TR_34_/L98H and TR_46_/Y121F/T289A). In addition to TRs, isolates with single point mutations developed and are widespread around the world (e.g. M220I, G54R). To date, azole-resistant isolates have been found on every continent except Antarctica. This review will summarize the epidemiology and prevalence of Azole-resistant *A. fumigatus* worldwide including the history of different mutations found and highlights important gaps as data are missing in several parts of the world.

## Introduction

*Aspergillus fumigatus* is a saprophytic mould, which is important for the degradation of organic matter. It is ubiquitous due to its abundant spore formation during the asexual reproduction cycle. *A. fumigatus* is a thermotolerant fungus, able to resist temperatures as high as 60°C and therefore has the ability to survive in very different environments.^1^ It is not primarily a pathogen for plants but it can cause a broad array of diseases in both humans and animals. *A. fumigatus* has evolved as an important cause of opportunistic fungal diseases in humans and is, according to newer estimations responsible for >50% of the invasive fungal disease burden globally.^2^ It is also listed in the World Health Organization (WHO) Fungal Priority Pathogen List (FPPL) in the critical group. In addition, *A. fumigatus* is the most frequent filamentous fungus colonizing the airways of patients with cystic fibrosis (CF), followed by *Scedosporium* spp.^3–6^ It can affect immunocompetent as well as immunocompromised individuals, but the presentation of the symptoms greatly depends on host defense and the spore load that was inhaled.^7^ The disease spectrum ranges from allergic conditions to invasive disease.^8^  *A. fumigatus* is able to colonize pre-existing cavities such as in the lung (forming aspergilloma), or the sinuses. Patients with cavitary lung diseases like tuberculosis or chronic obstructive pulmonary disease (COPD) can also develop chronic pulmonary aspergillosis (CPA), which results in occurrence of more cavities or ultimately pulmonary fibrosis.^9^ Allergic bronchopulmonary aspergillosis (ABPA) affects patients with cystic fibrosis (CF) or less frequently atopic patients.^10^ The allergic manifestation of *Aspergillus* can also lead to severe asthma with fungal sensitization (SAFS) or allergic rhinosinusitis. The most severe form of aspergillosis is invasive aspergillosis (IA), which affects mainly immunocompromised patients. It is primarily observed in patients with haematological malignancies like acute myelogenous leukemia (AML), those undergoing stem cell transplantation, patients with liver cirrhosis, those receiving glucocorticoid therapy and in patients who have undergone solid organ transplantation (SOT).^11^ Even with modern antifungal treatment, IA is still associated with high mortality.^12^ IA has also been observed after viral respiratory infections such as influenza and COVID-19 in otherwise immunocompetent individuals.^13^

### Treatment options for aspergillosis

*Aspergillus* disease is commonly treated with azoles,^14,15^ which inhibit the ergosterol biosynthesis pathway and interfere thereby with its integrity into the fungal cell membrane.^9^ Azoles are currently the only available anti-*Aspergillus* agents that are orally available and thus are important in long-term treatment, e.g. for CPA^16–19^ as well as for prophylaxis. However, two new oral antifungals await approval by the regulatory bodies (olorofim and manogepix), both showing activity against ARAf.

Due to *A. fumigatus’* intrinsic resistance to fluconazole, only four different azoles are in clinical use for the treatment of aspergillosis. All are available as both, oral and intravenous formulations. Itraconazole and voriconazole are often used for the treatment of CPA whereas voriconazole and isavuconazole are considered first-line therapy of IA.^14^ Posaconazole is used mainly for prophylaxis and salvage therapy in high-risk patients, e.g. with AML, haematopoietic cell transplant or SOT recipients.^14,15,20^ However, over time, acquired azole resistance has progressively evolved in *A. fumigatus* becoming an important clinical concern.^16^ Because of increasing cases of azole-resistant aspergillosis, international experts have recommended to consider abandoning azole monotherapy when resistance rates exceed 10%.^21^ In this context, azole-echinocandin combination therapy or liposomal AmB are considered as appropriate alternative treatment options.^9^ Therefore, determining azole resistance frequency in hospitals by e.g. regular susceptibility testing of clinical *A. fumigatus* isolates is pivotal to ensure the adequate management of patients with aspergillosis.

### Mechanisms underlying azole resistance in *Aspergillus fumigatus*

**Figure 1.**
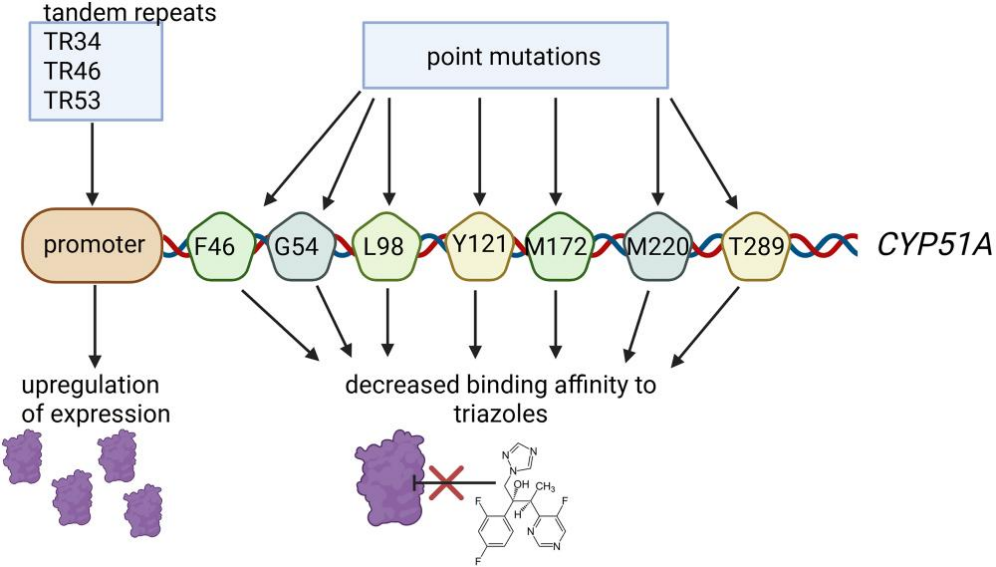
cyp51A-mediated azole resistance mechanisms involved in *Aspergillus fumigatus*. This figure highlights the role of tandem repeat duplications in the *CYP51A* promoter as well the main amino acid residues of the coding sequence involved in acquired resistance to azoles, when substituted.

Azole resistance can be caused by two different ways—by long-term exposure of patients to azoles (in-patient route) or by environmental exposure of *Aspergillus* to agricultural azoles (environmental route). In general, settings of actively reproducing *A. fumigatus* in the presence of azoles bears a risk of the development of mutations that confer azole resistance.^16^ Patient-evolved azole resistance is primarily observed in lung cavities like aspergilloma, e.g. in CPA and in CF patients.^5,9,22^ Mutations can be expected to arise over the course of invasive aspergillosis due to sub-lethal azole exposure. However, the hyphae of *A. fumigatus* contain millions of nuclei and a mutation in a limited number of these would be outnumbered by the other wild-type genes.^23^ Thus, asexual reproduction seems to be essential for developing phenotypic azole resistance, which is highly unlikely in patients with acute invasive aspergillosis, unless the infection progresses to a cavitary lesion.^24^ Clinical isolates of azole-resistant *A. fumigatus* show a broad variety of resistance mechanisms, which developed as a result of selection during azole therapy. Most isolates show point mutations in the *CYP51A* gene encoding one of two paralogs for lanosterol 14α-demethylase, a crucial enzyme in ergosterol biosynthesis. This enzyme is also the target of azole drugs^25^ and these point mutations leading to amino acid substitutions can directly affect azole binding. They occur at specific amino acid positions (e.g. G54 or M220), each differing in the level of resistance conferred to different azole compounds. Alternatively, an increased cyp51A expression,^26^ an increased efflux pump activity^27^ or other, non-cyp51A*-*mediated resistance mechanisms may be responsible for azole resistance.^28–32^ In contrast, azole-resistant strains found in the environment have distinct and specific signature mechanisms of resistance (e.g. TR_34_/L98H, TR_46_/Y121F/T289A). The first is the upregulation of the cyp51A expression by insertion of a tandem repeat (TR) of typically 34 or 46 base pairs in the promoter region. The second resistance mechanism is the acquisition of point mutations (in particular at codons 98, 121 and 289) in the gene which decreases the affinity of azoles for its target (Figure 1).^25^ Of note this emerging resistance may have been fueled by mutations in MSH6, a gene coding for a DNA-mismatch repair protein. Some variants (e.g. *msh6*-G233A) have been shown to lead to higher mutation rates and are frequently found in azole-resistant isolates.^33^ The environmental resistance route was found to be the dominating route for resistance in several studies.^34–37^ It has been hypothesized and further confirmed *in vitro*^38^ that resistance develops due to exposure of *A. fumigatus* to azole fungicides in the environment. This environmental route is also supported by the presence of resistance to non-azole agricultural fungicides in *A. fumigatus* environmental isolates.^39–41^ Fungicides such as difenoconazole, tebuconazole or epoxiconazole are chemically highly similar to clinically used triazoles and are therefore potentially responsible for the development of cross-resistance of *A. fumigatus* strains to medical azoles.^42^ While environmental azole application provides important selection pressure, the emergence of azole-resistant *Aspergillus fumigatus* (ARAf) likely is mainly restricted to selection hotspots.^43,44^ Vulnerable patient groups can develop IA after exposure to azole-resistant *A. fumigatus* conidia in the air, both inside hospitals^45^ and the home environment^46^ after which medical triazoles are no longer effective.^46^ This route was first shown to be an important source of azole resistance in the Netherlands, where 80%–90% of clinical isolates harbored one of the environmental resistance mechanisms.^9,34,47,48^ Of note, such strains have been rarely responsible for clonal outbreaks in hospital settings.^49^ Additionally, this resistance mechanism is found in isolates from animals.^50^ The presence of such dominant resistance mechanisms is difficult to explain if resistance had developed exclusively through treatment of patients with medical triazoles. Multiple resistance mechanisms would be expected in this case, because resistance would develop exclusively for isolates in each patient and a transfer of resistance mechanisms by transmission of strains between patients is also very unlikely in IA^47^ but possible between colonized CF patients.^51^ Breakthrough aspergillosis in patients on azole prophylaxis can also be explained via the environmental route: Patients inhale conidia of both azole-susceptible and azole-resistant strains and both can be detected by culture of sputum.^52^ Subsequently, the germination of resistant conidia in the lungs is favoured through azole prophylaxis and the patients can then develop IA with resistant strains.^47,53^ This is underlined by the fact that the fitness of *A. fumigatus* with cyp51A*-*related mutations may not be affected and the virulence of isolates with this resistance mechanism has been found to be similar to that of wild-type strains.^9,54^

*De novo* development of mutations is a rare event in *Aspergillus* and identical clones with TR mutations were found all over the world from environmental to clinical samples,^55,56^ indicating that dispersal gene flow is important for resistance spreading.^57^ In environmental habitats such as compost heaps, asexual and sexual reproduction occurs and increases the fungus’ ability to undergo genetic recombination and thereby overcome cellular stress due to azole exposure.^16^ Resistant isolates then compete with wild-type isolates in the field and persist in the environment because of a lack of fitness cost due to the mutations in the *CYP51A* gene.^54,58^ Presence of azoles and conditions favouring the reproduction of *A. fumigatus* are therefore needed for resistance development and define the key characteristics of resistance hotspots. Azole resistance hotspots have been described as ‘environments in which: (1) the physical, biotic and abiotic conditions facilitate the growth of the fungus and from which the fungus can spread; (2) this growth can take place for prolonged periods and the fungus can complete all the stages of its growth cycle; and (3) azoles are present, in different concentrations sufficient to select in populations and combinations’.^56^ Flower bulbs/flower bulb waste, green waste, compost heaps and wood chippings have therefore been defined as azole resistance hotspots.^59^ As explained above, there are two models for resistance development in *A. fumigatus:* (i) the environmental route, where the sexual reproduction cycle likely plays an important role and infections occur also in patients without apparent risk factors^60^ and (ii) the in-patient-route where mutations develop in patients with risk factors such as chronic infection/colonization during asexual reproduction.^22^

Point mutations in *CYP51A* and repeats in its promoter are the major mechanisms leading to azole resistance. Specific mutations have been associated with different azole susceptibility profiles. Isolates with mutations leading to G54E/R/W and P216L amino acid substitutions show high minimum inhibitory concentration (MIC) values for itraconazole and posaconazole whereas voriconazole is usually less affected. In contrast, mutations at codons G138 and G448 have been associated with a multi-azole-resistant phenotype.^61,62^ However, the most dominant azole-resistant phenotype is the environmental mutation TR_34_/L98H that leads to resistance to medical as well as agricultural azoles. In this combination, both alterations are necessary for azole resistance.^63^ Of note, strains displaying TR_46_/Y121F/T289A are highly resistant to voriconazole but resistance to itraconazole is variable. In addition, different point mutations were found worldwide that are associated with voriconazole resistance. Some of these are F291I, D262Y and F46Y/M172V/N248T/D255E/E427K.^64^ However, the latter is also found in azole-susceptible strains. This review will summarize the epidemiology and prevalence of ARAf worldwide including the history of different mutations found and highlights important gaps as data are missing in several parts of the world.

## Methods

The databases PubMed and Google Scholar were searched for articles on detection of ARAf and underlying mutations. ARAf were listed per country, first isolation date, and according to the main mutations found in CYP51A. Additionally, the latest ARAf prevalence reported were listed per country including the sampling size and the years of sample collection.

## Results and discussion

### The *A. fumigatus* complex

In the last 20 years, thanks to progress in taxonomy, new *Aspergillus* species have been identified, many of which being indistinguishable from each other by classical identification tools (macro and microscopic morphology). These new species, called ‘cryptic’ or ‘sibling’ species^65^ have led to the notion of ‘species complex’. In line with this, when using classical identification tools, *A. fumigatus* should be referred to as a ‘species complex’, which includes all closely related cryptic species. Besides *A. fumigatus sensu stricto* which is usually susceptible to triazole antifungals (except fluconazole) and to amphotericin B (AmB), the *Aspergillus fumigatus* complex includes several cryptic species with different antifungal susceptibility patterns (summarized in Table 1). *Aspergillus lentulus, A. fumigatiaffinis* and *A. udagawae* show high minimal inhibitory concentrations (MIC) for AmB. *A. lentulus* and *A. fumigatiaffinis* show also high MICs for medical triazoles, while *A. udagawae* displays only intermediate MICs for voriconazole and low MICs for itraconazole and posaconazole. *A. viridinutans* and *A. thermomutatus* (*A. pseudofischeri*) are resistant to azoles but susceptible to AmB. *A. oerlinghausenensis, A. hiratsukae* and *A. fumisynnematus* are susceptible to azoles and AmB.^65–71^ Here, we will focus on resistance mechanisms and resistance prevalence of *A. fumigatus sensu stricto* further referred to as *A. fumigatus*, only.

**Table 1.** Typical susceptibility pattern of cryptic species within the *Aspergillus fumigatus* complex

Cryptic species	*In vitro* susceptibility pattern		Cryptic species	*In vitro* susceptibility pattern	
*Aspergillus lentulus*	Amphotericin B	R	*Aspergillus thermomutatus / Aspergillus pseudofischeri*	Amphotericin B	S
	Itraconazole	R		Itraconazole	R
	Posaconazole	R		Posaconazole	R
	Voriconazole	R		Voriconazole	R
*Aspergillus fumigatiaffinis*	Amphotericin B	R	*Aspergillus hiratsukae*	Amphotericin B	S
	Itraconazole	R		Itraconazole	S
	Posaconazole	R		Posaconazole	S
	Voriconazole	R		Voriconazole	S
*Aspergillus udagawae*	Amphotericin B	R	*Aspergillus fumisynnematus*	Amphotericin B	S
	Itraconazole	S		Itraconazole	S
	Posaconazole	S		Posaconazole	S
	Voriconazole	S		Voriconazole	S
*Aspergillus viridinutans*	Amphotericin B	S	*Aspergillus oerlinghausenensis*	Amphotericin B	S
	Itraconazole	R		Itraconazole	S
	Posaconazole	R		Posaconazole	S
	Voriconazole	R		Voriconazole	S

Breakpoints for susceptibility testing of *A. fumigatus* according to EUCAST: susceptible (S): MIC ≤ 1 mg/l for AmB, ITR and VOR; MIC < 0.125 mg/l for POS. Resistant (R): MIC > 1 mg/l for AmB, ITR and VOR; MIC > 0.25 mg/l for POS.

### Global epidemiology of azole-resistant *A. fumigatus*

**Figure 2.**
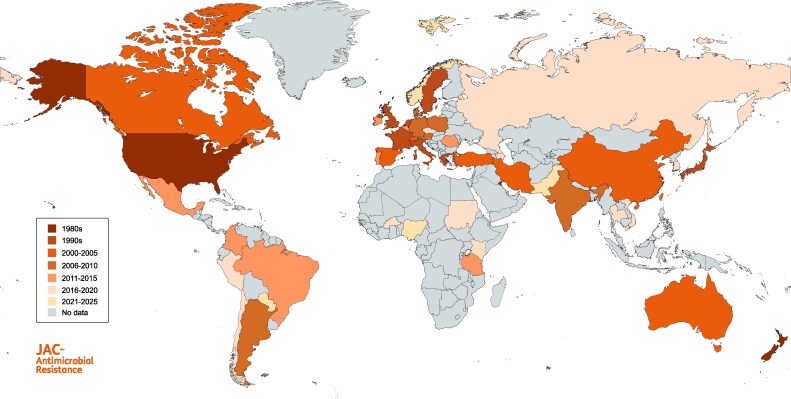
Azole-resistant *Aspergillus fumigatus* distribution around the world, sorted by year of first detection. *Data listed in Table 2. Map created with MapChart.net*.

**Figure 3.**
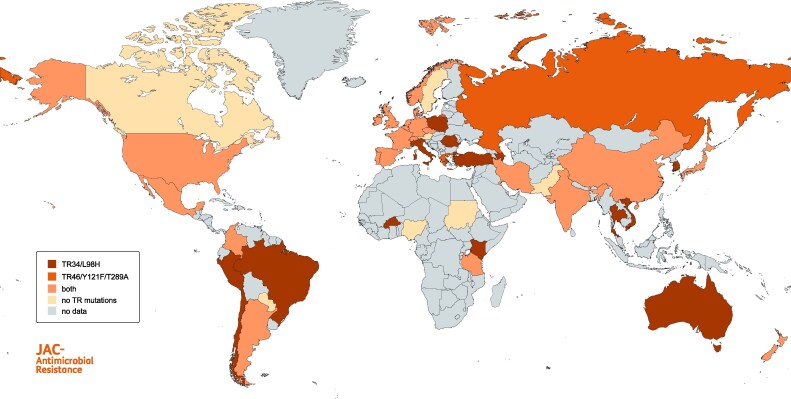
Global distribution of TR mutations in *Aspergillus fumigatus*.

**Figure 4.**
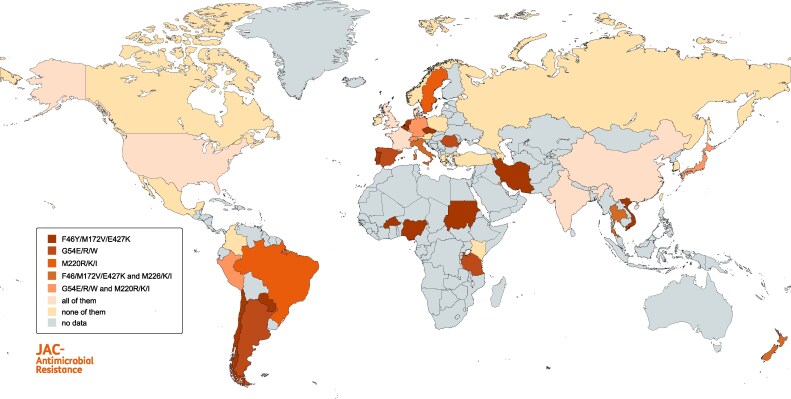
Global distribution of cyp51A mutations in *Aspergillus fumigatus*.

The first azole-resistant *A. fumigatus* strains were detected in the USA (California) in the late 1980s, all displaying resistance to itraconazole.^72^ The earliest azole-resistant isolates in Europe originate from 1997 and were found in The Netherlands, Sweden and France.^73–76^ One year later resistance was also documented in isolates from Italy and Japan.^77,78^ The resistant strains found in the Japanese study all harbored the G54 mutation.^78^ From then on, azole resistance was increasingly studied globally and thus reported in countries all over the world, including Canada,^79^ Brazil,^80^ China,^81^ Taiwan,^82^ Switzerland,^83^ Greece,^84^ the UK,^62^ Turkey^36^ and Australia^85^ (Table 2 and Figure 2). In 2007, Mellado *et al.* reported the discovery of the TR_34_/L98H resistance mechanism.^63^ This mechanism was subsequently detected worldwide (Figure 3) and later other TR resistance mechanisms including TR_46_/Y121F/T289A, TR_53_, and TR_120_^161^ were discovered. Mutations are still continuously evolving, leading to development of TR_34_/R65K/L98H, TR_34_/L98H/V242I/S297T/F495I and TR_46_/Y121F/V242I/T289A among others.^88,95,138,162^ Figures 3 and  4 show the distribution of the main mutations globally. The TR_34_/L98H mutation has been found on every continent except, in the absence of studies, Antarctica. It is mostly reported from Europe (71.1% of all reported environmental TR_34_/L98H isolates were found in Europe) and with the highest frequency in environmental ARAf isolates being reported from India (88.9% of Indian ARAf isolates harbor TR_34_/L98H), followed by Europe (77%), North America (58.8%), East Asia (37.4%), Middle East (37.1%), Africa (29.3%) and South America (4.3%).^59^ The other common environmental resistance mechanism TR_46_/Y121F/T289A has also been detected on every continent. Similarly, it is most prevalent in Europe (46.1% of all environmental TR_46_/Y121F/T289A isolates were reported in Europe), followed by South America (23.6%). This mutation has frequently been detected in South America (60% of all environmental ARAf), followed by East Asia (25.3%), North America (23.5%), Europe (12.3%), Africa (9.8%), India (9.5%) and Middle East (1.9%).^59^ Of note, only few studies from Africa are available and none of Antarctica, and frequencies reported are therefore difficult to compare. South America is the only region where TR_46_/Y121F/T289A seems more frequent than TR_34_/L98H. This could be a result of different crops grown in that area or different azole agricultural fungicides used.^59^ The TR_46_/Y121F/T289A mutants from The Netherlands are genetically highly related to isolates from India.^105^ This indicates that resistance traits can migrate very quickly around the world. Resistant isolates can also be imported into countries, through human activities. Intercountry transfer of ARAf isolates from The Netherlands to Ireland and Japan on plant bulbs^163,164^ has been reported previously, all showing TR mutations.^134^ The point mutations in G54 are less common than TR-mutations with an estimated worldwide prevalence of 1.8% among environmental ARAf isolates.^59^ They have been mostly found in Africa (13/22 environmental isolates), followed by Europe (8/22) and India (1/22).^59^ ARAf isolates harboring M172V are less common (0.9% prevalence) and mostly found in Europe (5/11 environmental isolates), North America (5/11) and East Asia (1/11).^59^ Taken together, G54 mutants are more frequently found in the East and M172V mutants in the West.

**Table 2.** Global prevalence of azole-resistant *Aspergillus fumigatus* and date of first sample from which ARAf was isolated

Country	Resistance mechanism	Isolation of oldest known ARAf strain	Source of first isolate	Year of report	Reference
North America					
Canada	Non-cyp51A-related	2002	C	2020	^79^
Mexico	TR_34_/L98H	2014–2017	C	2019	^86^
	TR_46_/Y121F/T289A	Not provided	E	2021	^87^
	Non-cyp51A-related	Not provided	E	2021	^87^
USA	Not determined	1980s	C	1997	^72^
	TR_34_/L98H	2010	C	2016	^88^
	TR_46_/Y121F/T289A	2008	C	2016	^88^
	Other cyp51A mutations	2001	C	2016	^88^
South America					
Argentina	TR_34_/L98H	2015	C	2020	^89^
	TR_46_/Y121F/T289A	2009	C	2018	^90^
	Other cyp51A mutations	2013	C	2017	^91^
	Non-cyp51A-related	2017	C	2020	^89^
Brazil	TR_34_/L98H	2015	C	2020	^92^
	Other cyp51A mutations	Not provided	E	2018	^93^
Chile	TR_34_/L98H	2017–2021	C	2024	^94^
	Other cyp51A mutations	2017–2022	C	2024	^94^
Colombia	TR_34_/L98H	2015	E	2016	^95–97^
	TR_46_/Y121F/T289A	2015	E	2016	^95–97^
	TR_53_	2015	E	2016	^95–97^
Paraguay	Other cyp51A mutations	Not provided	E	2021	^87^
Peru	TR_34_/L98H	2016–2017	C	2020	^98^
	Other cyp51A mutations	2016–2017	C	2020	^98^
Asia					
Azerbaijan	TR_34_/L98H	2017–2019	C	2025	^99^
	Non-cyp51A-related	2017–2019	C	2025	^99^
China	TR_34_/L98H	2008–2009	C	2011	^100^
	TR_46_/Y121F/T289A	2013	C	2015	^101^
	Other cyp51A mutations	2002–2003	C	2005	^81,102^
India	TR_34_/L98H	2008–2009	C	2012	^103^
	TR_46_/Y121F/T289A	2012–2013	E	2014	^104^
	Other cyp51A mutations	2013	C	2015	^105^
Iran	TR_34_/L98H	2003–2009	C + E	2013	^106,107^
	TR_46_/Y121F/T289A	2017–2018	E	2020	^108^
	Other cyp51A mutations	2016–2021	C + E	2022	^108,109^
	Non-cyp51A-related	2016–2021	C + E	2022	^108,109^
Japan	TR_34_/L98H	2016	C	2017	^110^
	TR_46_/Y121F/T289A	2013	C	2016	^111^
	Other cyp51A mutations	1998	C	2012	^78^
	Non-cyp51A-related	2000	C	2012	^78^
Korea	TR_34_/L98H	Not provided	C	2018	^112^
Kuwait	TR_34_/L98H	2011–2012	E	2014	^113^
	TR_46_/Y121F/T289A	2022	C	2023	^114^
Pakistan	Not determined	2021–2022	C + E	2022	^115^
Taiwan	Not determined	2003	C	2005	^82^
	TR_34_/L98H	2011–2014	C	2015	^116^
Thailand	TR_34_/L98H	Not provided	E	2016	^117^
	Other cyp51A mutations	Not provided	E	2016	^117,118^
Vietnam	TR_34_/L98H	2019	E	2021	^119^
	Other cyp51A mutations	2019	E	2021	^119^
	Non-cyp51A-related	2019	E	2021	^119^
Europe					
Austria	Not determined	2007–2012	C	2018	^120^
Belgium	TR_34_/L98H	2009–2011	C	2015	^121^
	TR_46_/Y121F/T289A	2012	C	2012	^122^
	Other cyp51A mutations	2020	Animals	2024	^50^
Czech Republic	TR_34_/L98H	2019–2021	C + E	2023	^123^
	TR_46_/Y121F/T289A	2019–2021	C + E	2023	^123^
	Other cyp51A mutations	2019–2021	C + E	2023	^123^
Denmark	TR_34_/L98H	2007	C	2011	^124^
	TR_46_/Y121F/T289A	2014	C	2014	^125^
	Other cyp51A mutations	2007	C	2011	^124^
	Non-cyp51A-related	2007	C	2011	^124^
France	Not determined	1997	C	1999	^73,74^
	TR_34_/L98H	2010–2011	C	2012	^45,126^
	TR_46_/Y121F/T289A	2013	C	2019	^127,128^
	Other cyp51A mutations	2010–2011	C	2012	^45,126^
	Non-cyp51A-related	2010–2011	C	2012	^45,126^
Germany	TR_34_/L98H	2012	C	2012	^129,130^
	TR_46_/Y121F/T289A	2012	C	2014	^131^
	Other cyp51A mutations	2010	C	2014	^131^
	Non-cyp51A-related	2012	C	2014	^132^
Greece	Not determined	1998–2009	C	2011	^84^
	TR_46_/Y121F/T289A	2016–2017	E	2020	^133^
Ireland	TR_34_/L98H	2014	E + C	2017	^19,134,135^
	TR_46_/Y121F/T289A	2014	E	2017	^134^
Italy	TR_34_/L98H	1998	C	2016	^77^
	Other cyp51A mutations	1999	C	2016	^77^
	Non-cyp51A-related	2001	C	2016	^77^
The Netherlands	Not determined	1990–2002	C	2002	^75^
	TR_34_/L98H	2002–2006	C	2007	^136,137^
	TR_46_/Y121F/T289A	2009–2011	C	2013	^137,138^
	Other cyp51A mutations	2002	C	2019	^137^
	Non-cyp51A-related	2002	C	2019	^137^
Norway	TR_34_/L98H	Not provided	E	2021	^139^
	TR_46_/Y121F/T289A	Not provided	E	2021	^139^
	Non-cyp51A-related	Not provided	E	2021	^139^
Poland	TR_34_/L98H	2007–2014	C	2015	^140^
Portugal	TR_34_/L98H	2010–2016	C	2018	^141^
	TR_46_/Y121F/T289A	2010–2016	C	2018	^141^
	Other cyp51A mutations	2010–2016	C	2018	^141^
	Non-cyp51A-related	2010–2016	C	2018	^141^
Romania	TR_34_/L98H	Not provided	E	2015	^142^
	Other cyp51A mutations	Not provided	E	2015	^142^
Russia	TR_46_/Y121F/T289A	2017	C	2024	^143^
Spain	TR_34_/L98H	2003	C	2013	^144^
	TR_46_/Y121F/T289A	2014	C	2015	^145^
	Other cyp51A mutations	2001	C	2011	^146^
Sweden	Other cyp51A mutations	1997	C	1997	^76^
Switzerland	Not determined	Not provided	C + E	2005	^83^
	TR_34_/L98H	20152017	C + E	2018	^147^
	TR_46_/Y121F/T289A	2019–2021	E	2023	^148^
	Other cyp51A mutations	2015	E	2018	^147^
Turkey	Not determined	2000	C	2015	^36^
	TR_34_/L98H	2000–2012	C	2015	^36^
	Non-cyp51A-related	2000–2012	C	2015	^36^
United Kingdom	TR_34_/L98H	1999–2007	C	2009	^62^
	TR_46_/Y121F/T289A	2016	C	2017	^149^
	Other cyp51A mutations	1999	C	2006	^150^
	Non-cyp51A-related	1999–2007	C	2009	^62^
Africa					
Burkina Faso	TR_34_/L98H	2021–2022	E	2024	^151^
	Other cyp51A mutations	2019	E	2021	^152^
Kenya	Not determined	Not provided	E	2017	^153^
	TR_34_/L98H	Not provided	C + E	2018	^154^
Nigeria	Other cyp51A mutations	Not provided	E	2021	^87^
Sudan	Not determined	2016–2019	C	2022	^155^
	Other cyp51A mutations	2015–2019	C	2023	^156^
Tanzania	TR_34_/L98H	Not provided	C + E	2014	^157,158^
	TR_46_/Y121F/T289A	Not provided	E	2014	^157^
	Other cyp51A mutations	Not provided	E	2015	^142^
Australia and Oceania					
Australia	TR_34_/L98H	2004	C	2015	^85^
	Other cyp51A mutations	2011	C	2015	^85^
New Zealand	TR_34_/L98H	2021–2024	C	2025	^159^
	TR_46_/Y121F/T289A	2021–2024	C	2025	^159^
	Other cyp51A mutations	2011	C	2021	^160^
	Non-cyp51A-related	2021–2024	C	2025	^159^

Source of isolate: C = clinical, E = environmental.

#### Europe

Most data on ARAf originate from Europe, where azole resistance has been intensively investigated in the last 20 years. In general, resistance rates in clinical and environmental isolates are particularly high in western Europe (ranging from 6% to 20%)^121^ and resistance started to rise in 2003 in clinical isolates.^165^ ARAf has been mostly detected in patients with chronic and/or allergic aspergillosis (prevalence from 15% to 20%).^62,166^ In 2015, a multicenter surveillance study determined the overall prevalence of ARAf in Europe to be around 3.2%, with ranges from 0% to 26% in different countries.^121^ However, the true prevalence in different countries is difficult to compare, since studies on ARAf differ greatly with respect to sampling (e.g. patient populations versus environment studies, sample size, location of study site, number of available studies), inclusion criteria, calculation (denominator) and isolation methods, among others.^167^ Nevertheless, the frequency of ARAf isolation has been included in this review for information purposes, but the numbers should be taken with a grain of salt, given the inherent limitations.

The lowest ARAf resistance rate was reported from Austria with 0.29% in clinical samples,^120^ Greece (1% in environmental samples)^133^ and Spain (1.15% in samples from environment or clinical).^168^ The highest resistance rate was reported in Romania on crop fields (24.2%),^142^ followed by Switzerland (18.6% in environmental samples).^148^ For an overview of the frequencies currently reported see Table 3. The environmental mutation TR_34_/L98H was first described in 2006 in samples from Spain and the Netherlands.^63,187^ TR_46_/Y121F/T289A was first reported in 2012/3 in samples from the Netherlands and Belgium dating to 2009 and 2011, respectively.^122,138^ An annual increase in itraconazole resistance from 1.7% to 6% was reported from 1994 to 2007 in clinical samples from The Netherlands.^47^ Interestingly, ARAf prevalence in France (although no national data are available) and Italy seems to be lower than in the Netherlands (2.1% and 6.6% in clinical samples respectively),^64,178^ even if ARAf had also been detected in these countries at the same time as in the Netherlands.^74,75,77^ In the UK, there has been an alarming increase in azole resistance reported in clinical isolates since 2004, with resistance increasing from 5% in 2004 to 14% in 2008 and later to 20% (2009).^62,165^ However, in the latest environmental studies, only 4.7% of *A. fumigatus* isolated from ambient air, showed resistance to medical azoles.^162^

**Table 3.** Latest ARAf frequency reported from different continents

Country	Latest ARAf frequency reported	Sample size (resistant isolates/tested isolates)	Source of isolates	Years of sample collection	Reference
North America					
Canada	0.3%	2/748	E	Not provided	^169^
Mexico	6.9%	7/102	E	Not provided	^87^
USA	26%	46/179	C + E	2015–2017	^170^
South America					
Argentina	14%	13/93	C	2016–2019	^89^
Brazil	1%	2/199	C	2014–2017	^92^
Chile	8.6%	2/23	C	2017–2021	^94^
Colombia	47.1%	15/34	E	Not provided	^96^
Paraguay	8.3%	3/36	E	Not provided	^87^
Peru	9.8%	6/61	E	Not provided	^87^
Asia					
Azerbaijan	10%	5/50	C + E	2017–2019	^99^
China	8.4%	7/83	C	2015–2019	^171^
India	25%	33/133	C	2017–2019	^172^
Iran	16.2%	78/483	C + E	2016–2021	^109^
Japan	1.6%	2/129	C	2011–2017	^173^
Korea	6.6%	6/91	C + E	2016–2018	^174^
Kuwait	4.5%	3/66	C + E	Not provided	^52^
Taiwan	5.9%	7/118	C	2016–2020	^175^
Thailand	27.4%	17/62	E	2021	^118^
Europe					
Belgium	2.6%	6/229	E	2020–2022	^176^
Czech Republic	6.3%	19/301	C	2019–2021	^123^
Denmark	4.2%	188/4538	E	2020–2022	^177^
France	2.1%	4/195	C	2017	^178^
Germany	1.8%	101/2888	C	2012–2016	^179^
Greece	1%	1/101	E	2016–2017	^133^
Italy	6.6%	19/286	C	2016–2018	^64^
The Netherlands	11.3%	16/142	Animals	2015–2020	^180^
Norway	3.7%	4/108	E	Not provided	^139^
Poland	4.1%	5/121	C	2009–2015	^181^
Portugal	8.1%	8/99	E	2018–2019	^182^
Romania	24.2%	23/95	E	2013–2014	^142^
Spain	1.2%	2/174	C + E	2019–2021	^168^
Switzerland	16.8%	19/113	E	2019–2021	^148^
Turkey	2.2%	19/850	C + E	2018–2019	^183^
United Kingdom	4.7%	111/2366	E	2018–2019	^184^
Africa					
Burkina Faso	3.23%	4/124	E	2021–2022	^151^
Kenya	22.2%	2/9	C	Not provided	^185^
Nigeria	2.2%	1/46	E	Not provided	^87^
Sudan	44.4%	4/9	C	2016–2019	^155^
Tanzania	26.4%	28/106	E	2013–2014	^142^
Australia and Oceania					
Australia	2%	3/148	C	2015–2017	^186^
New Zealand	10.2%	15/147	C	2021–2024	^159^

#### Asia

Data for ARAf are mostly available from south and east Asian countries. It was first detected in China^81^ and Taiwan^82^ in 2003 and 2005, respectively, but a retrospective study in Japan showed that ARAf has been present at least since 1998.^78^ The first TR-mutation, a TR_34_/L98H mutation, was documented in 2005 in Iran.^106^ Interestingly, a point mutation in G54 seems to be more prevalent in Asia than TR-mutations. In Japan, China and India, G54 mutations are the most common mutation reported to date,^171–173^ and are also found next to TR_34_/L98H in the environment of Thailand.^117^ Considering the little available data, the frequency of ARAf is difficult to assess, but could be slightly higher compared to Europe. A recently published review showed an overall resistance prevalence of 4% in clinical isolates and 14% in environmental isolates.^188^ The lowest resistance frequency reported in Asia was from Japan (1.55% in clinical samples).^173^ Along with Kuwait, which showed a frequency of 4.7% in both clinical and environmental samples, these are the only two reported frequencies below 5% on this continent.^52^ The ARAf prevalence of the other countries range from 5.9% in clinical samples from Taiwan^175^ to 16.1% in environmental and clinical samples in Iran,^109^ 25% in clinical samples in India^172^ and 27.4% in environmental samples from Thailand.^118^

#### North America

The first ARAf isolate was documented in the USA in the late 1980s^72^ and the first TR-mutations were present as early as 2008.^88^ Later, different resistance frequencies were reported. Two passive surveillance studies demonstrated a prevalence of 5% ARAf and 1.4% both in clinical isolates^189,190^ and one environmental study performed in the south-eastern region of the USA reported a prevalence of 19%.^191^ The latest study on clinical and environmental *A. fumigatus* isolates reported an alarmingly high prevalence of 26%.^170^ In contrast, resistance rates are considerably lower in bordering Mexico and Canada. In Mexico, the first ARAf was found in a retrospective study performed on clinical isolates collected between 2014 and 2017.^86^ The latest resistance rate of environmental samples was 6.9%, reaching from 3.7% in Mexico City to 11.6% in Guanajuato, a region with intense agricultural activities.^87^ In Canada, the first ARAf isolate originated from 2002 and was discovered in a retrospective environmental study.^79^ However, the latest resistance rate (also an environmental study) was extremely low (0.3%).^169^

#### South America

The distribution of ARAf in South America is similar to the distribution in Europe with resistance rates in clinical samples ranging from 1% in Brazil^92^ to 14% in Argentina.^89^ Of note, resistance frequencies of 47.1% and 44.1% were reported in two environmental studies in Colombia.^95,96^ However, the isolates were mainly collected from flower fields, so this value seems to be more representative for hotspots with elevated fungicide use. The first ARAf isolate for South America was detected in Brazil in a retrospective clinical study with samples from 2006 to 2013,^80^ followed by a TR_46_/Y121F/T289A mutant discovered in 2009 in Argentina from clinical isolates.^90^ Overall, several TR_34_/L98H variants with additional point mutations have been found on this continent including TR_34_/L98H/S297T, TR_34_/L98H/S297T/F495I and TR_34_/R65K/L98H/S297T.^87,89,92^ In Paraguay, no TR-mutation have been reported to date. The only mutations found were F46Y/M172V/E427K and F46Y/M172V/N248T/D255E/E427K.^87^

#### Africa

In Africa, ARAf isolates were first detected in Tanzania between 2013 and 2014^142^ and later also in Sudan,^155^ Kenya,^153^ Burkina Faso^152^ and Nigeria.^87^ Based on the available data, resistance rates in East Africa seem to be considerably higher than in West Africa. ARAf rates of 22.2% and 26.4% in clinical isolates were reported in Kenya and Tanzania respectively,^142,185^ and 44.4% in Sudan in environmental samples.^155^ However, the sample size in these studies were small (see Table 3) so these frequencies should be interpreted with caution. Extensive use of fungicides in the flower and horticultural industry in Kenya and the farming regions in Tanzania could explain the high values in these countries (32% in Kenya^153^ and 26,4% in Tanzania^142^ in environment).^154,192^ In contrast, the resistance rates reported from West Africa are quite low. Only 3.23% and 2.2% environmental ARAf isolates were documented in Burkina Faso and Nigeria, respectively.^87,151^ In addition, TR-mutations were only found in Kenya, Tanzania and Burkina Faso. In Tanzania, TR_34_/L98H and TR_46_/Y121F/T289A were detected, whereas in Kenya and Burkina Faso, only TR_34_/L98H was found.^142,151,154^

#### Australia and Oceania

Only a limited number of studies have been conducted on this continent. In 2004, the first ARAf clinical isolate—exhibiting TR_34_/L98H mutation—was found in Australia.^85^ The latest resistance frequency reported was 2% in clinical isolates.^186^ In New Zealand, the first ARAf was isolated in 2018.^160^ To date, several mutations in cyp51A like G54E/W, F46Y/G89E/M172V/E427K/G448S and TR_34_ and TR_46_ strains were found in this country.^159^ The latest study from New Zealand found 10.2% of clinical isolates to be azole resistant.^159^

## Conclusion

ARAf are present globally. Fourteen out of 41 countries (34%) in which prevalence studies have been conducted and published, reported a resistance rate over 10%, which is the number above which experts recommend to consider switching from azoles to amphotericin B or combination therapy as first-line treatment of IA.^16,21^ However, it is important to take regional variations in prevalence into account, especially in larger countries. Therefore, it is indispensable to monitor ARAf prevalence and collect more regional epidemiological data, employing harmonized study protocols.

The most prevalent mutations are unambiguously TR mutations, with TR_34_ being more prevalent than TR_46_ in all continents except for South America. Other point mutations in cyp51A are also widespread but not equally distributed around the globe. Among these, mutations in G54 are more frequently found in the East while mutations containing M172V point mutation seem more prevalent in the West. Overall this review highlights important gaps as data are missing in several parts of the world, especially Africa and Antarctica. Additionally, there is a need for long term national and international surveillance programs and agreement on standardized methods and protocols. Data from some countries are almost ten years old and might not be representative of today's epidemiology, as previous studies demonstrated that resistance can rapidly increase. Taken together, ARAf has emerged globally and its frequency should be further monitored in the environment and in clinical samples.

Besides surveillance, further steps have to be taken to limit the further increase of azole resistance. This requires a One Health approach, including human, animal and agricultural consumption of antifungals. For the latter, the prudent use of azole fungicides should be promoted, minimizing exposure of *A. fumigatus* to azoles. Before the approval of new fungicides, the development of cross resistance to medical antifungals has to be analyzed and considered in the approval process. Additionally, the management of agricultural and clinical waste/sewage have to be further improved. While the inpatient development of azole resistance plays only a minor role for the dissemination of ARAf, antifungal stewardship efforts should be increased to reduce the exposure of *A. fumigatus* and other fungi to antifungals. The prudent use of antifungals in medicine can be further strengthened by improving diagnostic tools before antifungal treatment is initiated and by including additional diagnostic methods in the standard approach in case of a suspicion of IA, e.g. *Aspergillus* PCR in combination with galactomannan antigen detection and culture from bronchoalveolar lavage. Potentially, leveraging artificial intelligence could aid in decision making, e.g. by improved interpretation of laboratory and radiological findings, leading to a more targeted antifungal use.

Besides stewardship efforts and surveillance in agriculture and medicine, new antifungals need to be developed, in particular with new cellular targets.

## Transparency declarations

Declaration of interests OAC declares grants from the German Federal Ministry of Education and Research (BMBF), Cidara, the German Center for Infection Research (DZIF), EU Directorate-General Research and Innovation, F2G, Gilead, MedPace, MSD, Mundipharma, Octapharma, Pfizer, and Scynexis; consulting fees from AbbVie, AiCuris, Basilea, Biocon, Cidara, Seqirus, Gilead, GSK, IQVIA, Janssen, Matinas, MedPace, Menarini, Molecular Partner, MSGERC, Mundipharma, Noxxon, Octapharma, Pardes, Partner Therapeutics, Pfizer, PSI, Scynexis, and Seres; honoraria from Abbott, AbbVie, Akademie für Infektionsmedizin, Al-Jazeera Pharmaceuticals–Hikma, amedes, AstraZeneca, Deutscher Ärzteverlag, Gilead, GSK, Grupo Biotoscana–United Medical–Knight, Ipsen Pharma, Medscape–WebMD, MedUpdate, MSD, Moderna, Mundipharma, Noscendo, Paul-Martini-Stiftung, Pfizer, Sandoz, Seqirus, Shionogi, streamedup!, Touch Independent, and Vitis; payment for expert testimony from Cidara; and board participation from Boston Strategic Partners, Cidara, IQVIA, Janssen, MedPace, PSI, Pulmocide, Shionogi, The Prime Meridian Group, and Vedanta Biosciences. OK declares research grants from the EU, German Research Foundation (DFG), BMBF, and BMG; consulting fees paid to his institution from Mundipharma; honoraria paid to his institution from Gilead, Pfizer, and Fujifilm Wako; receipt of microbiology media for evaluation from Mast Diagnostika; and reports receipt of chemical substances (antifungal drugs) for susceptibility testing. FM has received funds for participating in educational activities organized on behalf of Gilead, and Mundipharm and research funds from Pfizer, Gilead, Mundipharma, Agence Nationale de la Recherche and CAPES-COFECUB. AH declares honoraria from Shionogi, bioMérieux and research support from Bruker. All other authors declare no competing interests.

## References

[1] Kwon-Chung KJ, Sugui JA. *Aspergillus fumigatus*—what makes the species a ubiquitous human fungal pathogen? PLoS Pathog 2013; 9: e1003743. 10.1371/journal.ppat.1003743 24348239 PMC3857757

[2] Denning DW . Global incidence and mortality of severe fungal disease. Lancet Infect Dis 2024; 24: e428–38. 10.1016/S1473-3099(23)00692-8 38224705

[3] Engel TGP, Slabbers L, De Jong C et al Prevalence and diversity of filamentous fungi in the airways of cystic fibrosis patients—A Dutch, multicentre study. J Cyst Fibros 2019; 18: 221–6. 10.1016/j.jcf.2018.11.012 30514613

[4] Felton IC, Simmonds NJ. Aspergillus and cystic fibrosis. Curr Opin Pulm Med 2014; 20: 632–8. 10.1097/MCP.0000000000000106 25229669

[5] Hamprecht A, Morio F, Bader O et al Azole resistance in *Aspergillus fumigatus* in patients with cystic fibrosis: a matter of concern? Mycopathologia 2018; 183: 151–60. 10.1007/s11046-017-0162-4 28653258

[6] Pihet M, Carrere J, Cimon B et al Occurrence and relevance of filamentous fungi in respiratory secretions of patients with cystic fibrosis—a review. Med Mycol 2009; 47: 387–97. 10.1080/13693780802609604 19107638

[7] Latgé J-P . *Aspergillus fumigatus* and Aspergillosis. Clin Microbiol Rev 1999; 12: 310–50. 10.1128/CMR.12.2.310 10194462 PMC88920

[8] Kosmidis C, Denning DW. The clinical spectrum of pulmonary aspergillosis. Thorax 2015; 70: 270–7. 10.1136/thoraxjnl-2014-206291 25354514

[9] Meis JF, Chowdhary A, Rhodes JL et al Clinical implications of globally emerging azole resistance in *Aspergillus fumigatus*. Philos Trans R Soc B Biol Sci 2016; 371: 20150460. 10.1098/rstb.2015.0460 PMC5095539 28080986

[10] Agarwal R, Sehgal IS, Muthu V et al Revised ISHAM-ABPA working group clinical practice guidelines for diagnosing, classifying and treating allergic bronchopulmonary aspergillosis/mycoses. Eur Respir J 2024; 63: 2400061. 10.1183/13993003.00061-2024 38423624 PMC10991853

[11] Latgé JP, Chamilos G. *Aspergillus fumigatus* and aspergillosis in 2019. Clin Microbiol Rev 2020; 33: e00140-18. 10.1128/CMR.00140-18 PMC6860006 31722890

[12] Bretagne S, Sitbon K, Desnos-Ollivier M et al Active surveillance program to increase awareness on invasive fungal diseases: the French RESSIF network (2012 to 2018). mBio 2022; 13: e0092022. 10.1128/mbio.00920-22 35499498 PMC9239099

[13] Koehler P, Bassetti M, Chakrabarti A et al Defining and managing COVID-19-associated pulmonary aspergillosis: the 2020 ECMM/ISHAM consensus criteria for research and clinical guidance. Lancet Infect Dis 2021; 21: e149–62. 10.1016/S1473-3099(20)30847-1 33333012 PMC7833078

[14] Ullmann AJ, Aguado JM, Arikan-Akdagli S et al Diagnosis and management of Aspergillus diseases: executive summary of the 2017 ESCMID-ECMM-ERS guideline. Clin Microbiol Infect Dis 2018; 24 Suppl 1: e1–38. 10.1016/j.cmi.2018.01.002 29544767

[15] Patterson TF, Thompson GR, Denning DW et al Practice guidelines for the diagnosis and management of aspergillosis: 2016 update by the Infectious Diseases Society of America. Clin Infect Dis 2016; 63: e1–60. 10.1093/cid/ciw444 27365388 PMC4967602

[16] Verweij PE, Chowdhary A, Melchers WJG et al Azole resistance in aspergillus fumigatus: can we retain the clinical use of mold-active antifungal azoles? Clin Infect Dis 2016; 62: 362–8. 10.1093/cid/civ885 26486705 PMC4706635

[17] Meijer EFJ, Dofferhoff ASM, Hoiting O et al COVID-19-associated pulmonary aspergillosis: a prospective single-center dual case series. Mycoses 2021; 64: 457–64. 10.1111/myc.13254 33569857 PMC7986084

[18] Meijer EFJ, Dofferhoff ASM, Hoiting O et al Azole-resistant COVID-19-associated pulmonary aspergillosis in an immunocompetent host: a case report. J Fungi 2020; 6: 79. 10.3390/jof6020079 PMC7344504 32517166

[19] Talento AF, Dunne K, Murphy N et al Post-influenzal triazole-resistant aspergillosis following allogeneic stem cell transplantation. Mycoses 2018; 61: 570–5. 10.1111/myc.12770 29570855

[20] Denning DW, Cadranel J, Beigelman-Aubry C et al Chronic pulmonary aspergillosis: rationale and clinical guidelines for diagnosis and management. Eur Respir J 2016; 47: 45–68. 10.1183/13993003.00583-2015 26699723

[21] Verweij PE, Ananda-Rajah M, Andes D et al International expert opinion on the management of infection caused by azole-resistant *Aspergillus fumigatus*. Drug Resist Updat 2015; 21–22: 30–40. 10.1016/j.drup.2015.08.001 26282594

[22] Verweij PE, Zhang J, Debets AJM et al In-host adaptation and acquired triazole resistance in *Aspergillus fumigatus*: a dilemma for clinical management. Lancet Infect Dis 2016; 16: e251–60. 10.1016/S1473-3099(16)30138-4 27638360

[23] Verweij PE, Snelders E, Kema GH et al Azole resistance in *Aspergillus fumigatus*: a side-effect of environmental fungicide use? Lancet Infect Dis 2009; 9: 789–95. 10.1016/S1473-3099(09)70265-8 19926038

[24] Lamb BC, Mandaokar S, Bahsoun B et al Differences in spontaneous mutation frequencies as a function of environmental stress in soil fungi at ‘”Evolution Canyon,”‘ Israel. Proc Natl Acad Sci U S A 2008; 105: 5792–6. 10.1073/pnas.0801995105 18401030 PMC2311348

[25] Chowdhary A, Sharma C, Meis JF. Azole-resistant aspergillosis: epidemiology, molecular mechanisms, and treatment. J Infect Dis 2017; 216: S436–44. 10.1093/infdis/jix210 28911045

[26] Camps SMT, Rijs AJMM, Klaassen CHW et al Molecular epidemiology of *Aspergillus fumigatus* isolates harboring the TR34/L98H azole resistance mechanism. J Clin Microbiol 2012; 50: 2674–80. 10.1128/JCM.00335-12 22675126 PMC3421523

[27] Fraczek MG, Bromley M, Buied A et al The cdr1B efflux transporter is associated with non-cyp51a-mediated itraconazole resistance in *Aspergillus fumigatus*. J Antimicrob Chemother 2013; 68: 1486–96. 10.1093/jac/dkt075 23580559

[28] Hagiwara D, Arai T, Takahashi H et al Non-cyp51A azole-resistant *Aspergillus fumigatus* isolates with mutation in HMG-CoA reductase. Emerg Infect Dis 2018; 24: 1889–97. 10.3201/eid2410.180730 30226177 PMC6154143

[29] Rybak JM, Ge W, Wiederhold NP et al Mutations in *hmg1*, challenging the paradigm of clinical triazole resistance in *Aspergillus fumigatus*. mBio 2019; 10: e00437-19. 10.1128/mBio.00437-19 30940706 PMC6445940

[30] Camps SMT, Dutilh BE, Arendrup MC et al Discovery of a hapE mutation that causes azole resistance in *Aspergillus fumigatus* through whole genome sequencing and sexual crossing. PLoS One 2012; 7: e50034. 10.1371/journal.pone.0050034 23226235 PMC3511431

[31] Xiong Q, Hassan SA, Wilson WK et al Cholesterol import by *Aspergillus fumigatus* and its influence on antifungal potency of sterol biosynthesis inhibitors. Antimicrob Agents Chemother 2005; 49: 518–24. 10.1128/AAC.49.2.518-524.2005 15673727 PMC547240

[32] Willger SD, Puttikamonkul S, Kim KH et al A sterol-regulatory element binding protein is required for cell polarity, hypoxia adaptation, azole drug resistance, and virulence in *Aspergillus fumigatus*. PLoS Pathog 2008; 4: e1000200. 10.1371/journal.ppat.1000200 18989462 PMC2572145

[33] Bottery MJ, van Rhijn N, Chown H et al Elevated mutation rates in multi-azole resistant *Aspergillus fumigatus* drive rapid evolution of antifungal resistance. Nat Commun 2024; 15: 10654. 10.1038/s41467-024-54568-5 39681549 PMC11649685

[34] Song Y, Buil JB, Rhodes J et al Triazole-resistant *Aspergillus fumigatus* in the Netherlands between 1994 and 2022: a genomic and phenotypic study. Lancet Microbe 2025; 6: 101114. 10.1016/j.lanmic.2025.101114 40651492

[35] Cuypers L, Aerts R, Van de Gaer O et al Doubling of triazole resistance rates in invasive aspergillosis over a 10-year period, Belgium, 1 April 2022 to 31 March 2023. Euro Surveill 2025; 30: 2400559. 10.2807/1560-7917.ES.2025.30.18.2400559 40341104 PMC12066980

[36] Özmerdiven GE, Ak S, Ener B et al First determination of azole resistance in *Aspergillus fumigatus* strains carrying the TR34/L98H mutations in Turkey. J Infect Chemother 2015; 21: 581–6. 10.1016/j.jiac.2015.04.012 26048062

[37] Vermeulen E, Maertens J, De Bel A et al Nationwide surveillance of azole resistance in Aspergillus diseases. Antimicrob Agents Chemother 2015; 59: 4569–76. 10.1128/AAC.00233-15 25987612 PMC4505291

[38] Ren J, Jin X, Zhang Q et al Fungicides induced triazole-resistance in *Aspergillus fumigatus* associated with mutations of TR46/Y121F/T289A and its appearance in agricultural fields. J Hazard Mater 2017; 326: 54–60. 10.1016/j.jhazmat.2016.12.013 27987450

[39] European Food Safety Authority (EFSA), European Centre for Disease Prevention and Control (ECDC), European Chemicals Agency (ECHA) et al Impact of the use of azole fungicides, other than as human medicines, on the development of azole-resistant *Aspergillus* spp. EFSA J 2025; 23: e9200. 10.2903/j.efsa.2025.9200 39886075 PMC11780318

[40] Kanellopoulos SG, Snelders E. Moving beyond multi-triazole to multi-fungicide resistance: broader selection of drug resistance in the human fungal pathogen *Aspergillus fumigatus*. PLoS Pathog 2025; 21: e1012851. 10.1371/journal.ppat.1012851 39928648 PMC11809870

[41] Hartuis S, Robert M, Chouaki T et al Multi-fungicide resistance in both clinical and environmental isolates of the human fungal pathogen *Aspergillus fumigatus*. Med Mycol 2025: myaf110. 10.1093/mmy/myaf110 41334822

[42] Chowdhary A, Kathuria S, Xu J et al Emergence of azole-resistant *Aspergillus fumigatus* strains due to agricultural azole use creates an increasing threat to human health. PLoS Pathog 2013; 9: e1003633. 10.1371/journal.ppat.1003633 24204249 PMC3812019

[43] Schoustra SE, Debets AJM, Rijs AJMM et al Environmental hotspots for azole resistance selection of *Aspergillus fumigatus*, the Netherlands. Emerg Infect Dis 2019; 25: 1347–53. 10.3201/eid2507.181625 31211684 PMC6590754

[44] Barber AE, Riedel J, Sae-Ong T et al Effects of agricultural fungicide use on *Aspergillus fumigatus* abundance, antifungal susceptibility, and population structure. mBio 2020; 11: e02213-20. 10.1128/mBio.02213-20 33234685 PMC7701986

[45] Burgel PR, Baixench MT, Amsellem M et al High prevalence of azole-resistant *Aspergillus fumigatus* in adults with cystic fibrosis exposed to itraconazole. Antimicrob Agents Chemother 2012; 56: 869–74. 10.1128/AAC.05077-11 22123701 PMC3264284

[46] Lavergne R-A, Chouaki T, Hagen F et al Home environment as a source of life-threatening azole-resistant *Aspergillus fumigatus* in immunocompromised patients. Clin Infect Dis 2017; 64: 76–8. 10.1093/cid/ciw664 27682064

[47] Snelders E, Van Der Lee HAL, Kuijpers J et al Emergence of azole resistance in *Aspergillus fumigatus* and spread of a single resistance mechanism. PLoS Med 2008; 5: 1629–37. 10.1371/journal.pmed.0050219 PMC2581623 18998768

[48] van der Linden JWM, Snelders E, Kampinga GA et al Clinical implications of azole resistance in *Aspergillus fumigatus*, the Netherlands, 2007–2009. Emerg Infect Dis 2011; 17: 1846–54. 10.3201/eid1710.110226 22000354 PMC3311118

[49] Joste V, Delouis M, Mouhajir A et al Genomic investigation of an antifungal-resistant *Aspergillus fumigatus* outbreak in a French hospital. Med Mycol 2025; 63: myaf012. 10.1093/mmy/myaf012 39924166

[50] Debergh H, Haesendonck R, Botteldoorn N et al Pan-azole resistance in clinical *Aspergillus fumigatus* isolates carrying TR34/L98H from birds and mammals in Belgium. One Health 2024; 19: 100907. 10.1016/j.onehlt.2024.100907 39430230 PMC11490837

[51] Engel TGP, Erren E, Vanden Driessche KSJ et al Aerosol transmission of *Aspergillus fumigatus* in cystic fibrosis patients in the Netherlands. Emerg Infect Dis 2019; 25: 797–9. 10.3201/eid2504.181110 30882308 PMC6433022

[52] Ahmad S, Joseph L, Hagen F et al Concomitant occurrence of itraconazole-resistant and -susceptible strains of *Aspergillus fumigatus* in routine cultures. J Antimicrob Chemother 2015; 70: 412–5. 10.1093/jac/dku410 25326091

[53] Cowen LE . Predicting the emergence of resistance to antifungal drugs. FEMS Microbiol Lett 2001; 204: 1–7. 10.1111/j.1574-6968.2001.tb10853.x 11682169

[54] Chen S, Zhu G, Lin H et al Variability in competitive fitness among environmental and clinical azole-resistant *Aspergillus fumigatus* isolates. mBio 2024; 15: e0026324. 10.1128/mbio.00263-24 38407058 PMC11005360

[55] Snelders E, Camps SMT, Karawajczyk A et al Triazole fungicides can induce cross-resistance to medical triazoles in *Aspergillus fumigatus*. PLoS One 2012; 7: e31801. 10.1371/journal.pone.0031801 22396740 PMC3291550

[56] Verweij PE, Lucas JA, Arendrup MC et al The one health problem of azole resistance in *Aspergillus fumigatus*: current insights and future research agenda. Fungal Biol Rev 2020; 34: 202–14. 10.1016/j.fbr.2020.10.003

[57] Sewell TR, Zhu J, Rhodes J et al Nonrandom distribution of azole resistance across the global population of *Aspergillus fumigatus*. mBio 2019; 10: e00392-19. 10.1128/mBio.00392-19 31113894 PMC6529631

[58] Mavridou E, Meletiadis J, Jancura P et al Composite survival index to compare virulence changes in azole-resistant *Aspergillus fumigatus* clinical isolates. PLoS One 2013; 8: e72280. 10.1371/journal.pone.0072280 23991080 PMC3753310

[59] Burks C, Darby A, Gómez Londoño L et al Azole-resistant *Aspergillus fumigatus* in the environment: identifying key reservoirs and hotspots of antifungal resistance. PLoS Pathog 2021; 17: e1009711. 10.1371/journal.ppat.1009711 34324607 PMC8321103

[60] Zhang J, Snelders E, Zwaan BJ et al A novel environmental azole resistance mutation in *Aspergillus fumigatus* and a possible role of sexual reproduction in its emergence. mBio 2017; 8: e00791-17. 10.1128/mBio.00791-17 28655821 PMC5487732

[61] Dudakova A, Spiess B, Tangwattanachuleeporn M et al Molecular tools for the detection and deduction of azole antifungal drug resistance phenotypes in Aspergillus species. Clin Microbiol Rev 2017; 30: 1065–91. 10.1128/CMR.00095-16 28903985 PMC5608879

[62] Howard SJ, Cerar D, Anderson MJ et al Frequency and evolution of azole resistance in *Aspergillus fumigatus* associated with treatment failure. Emerg Infect Dis 2009; 15: 1068–76. 10.3201/eid1507.090043 19624922 PMC2744247

[63] Mellado E, Garcia-Effron G, Alcázar-Fuoli L et al A new *Aspergillus fumigatus* resistance mechanism conferring in vitro cross-resistance to azole antifungals involves a combination of cyp51A alterations. Antimicrob Agents Chemother 2007; 51: 1897–904. 10.1128/AAC.01092-06 17371828 PMC1891382

[64] Prigitano A, Esposto MC, Grancini A et al Azole resistance in Aspergillus isolates by different types of patients and correlation with environment—an Italian prospective multicentre study (ARiA study). Mycoses 2021; 64: 528–36. 10.1111/myc.13241 33438319

[65] Alastruey-Izquierdo A, Alcazar-Fuoli L, Cuenca-Estrella M. Antifungal susceptibility profile of cryptic species of aspergillus. Mycopathologia 2014; 178: 427–33. 10.1007/s11046-014-9775-z 24972670

[66] Alcazar-Fuoli L, Mellado E, Alastruey-Izquierdo A et al *Aspergillus* section *Fumigati*: antifungal susceptibility patterns and sequence-based identification. Antimicrob Agents Chemother 2008; 52: 1244–51. 10.1128/AAC.00942-07 18212093 PMC2292508

[67] Balajee SA, Gribskov J, Brandt M et al Mistaken identity: neosartorya pseudofischeri and its anamorph masquerading as *Aspergillus fumigatus*. J Clin Microbiol 2005; 43: 5996–9. 10.1128/JCM.43.12.5996-5999.2005 16333088 PMC1317194

[68] Montenegro G, Sánchez Puch S, Jewtuchowicz VM et al Phenotypic and genotypic characterization of *Aspergillus lentulus* and *Aspergillus fumigatus* isolates in a patient with probable invasive aspergillosis. J Med Microbiol 2009; 58: 391–5. 10.1099/jmm.0.005942-0 19208894

[69] Sugui JA, Vinh DC, Nardone G et al Neosartorya udagawae (*Aspergillus udagawae*), an emerging agent of Aspergillosis: how different is it from *Aspergillus fumigatus*? J Clin Microbiol 2010; 48: 220–8. 10.1128/JCM.01556-09 19889894 PMC2812273

[70] Vinh DC, Shea YR, Jones PA et al Chronic invasive aspergillosis caused by *Aspergillus viridinutans*. Emerg Infect Dis 2009; 15: 1292–4. 10.3201/eid1508.090251 19751595 PMC2815978

[71] Houbraken J, Weig M, Groß U et al *Aspergillus oerlinghausenensis*, a new mould species closely related to *A. fumigatus*. FEMS Microbiol Lett 2016; 363: fnv236. 10.1093/femsle/fnv236 26667219

[72] Denning DW, Venkateswarlu K, Oakley KL et al Itraconazole resistance in *Aspergillus fumigatus*. Antimicrob Agents Chemother 1997; 41: 1364–8. 10.1128/AAC.41.6.1364 9174200 PMC163916

[73] Dannaoui E, Borel E, Monier MF et al Acquired itraconazole resistance in *Aspergillus fumigatus*. J Antimicrob Chemother 2001; 47: 333–40. 10.1093/jac/47.3.333 11222566

[74] Dannaoui E, Persat F, Monier MF et al In-vitro susceptibility of *Aspergillus* spp. isolates to amphotericin B and itraconazole. J Antimicrob Chemother 1999; 44: 553–5. 10.1093/jac/44.4.553 10588320

[75] Verweij PE, Te Dorsthorst DTA, Rijs AJMM et al Nationwide survey of in vitro activities of itraconazole and voriconazole against clinical *Aspergillus fumigatus* isolates cultured between 1945 and 1998. J Clin Microbiol 2002; 40: 2648–50. 10.1128/JCM.40.7.2648-2650.2002 12089298 PMC120540

[76] Chryssanthou E . In vitro susceptibility of respiratory isolates of *Aspergillus* species to itraconazole and amphotericin B. acquired resistance to itraconazole. Scand J Infect Dis 1997; 29: 509–12. 10.3109/00365549709011864 9435042

[77] Lazzarini C, Esposto MC, Prigitano A et al Azole resistance in *Aspergillus fumigatus* clinical isolates from an Italian culture collection. Antimicrob Agents Chemother 2016; 60: 682–5. 10.1128/AAC.02234-15 26552980 PMC4704201

[78] Tashiro M, Izumikawa K, Hirano K et al Correlation between triazole treatment history and susceptibility in clinically isolated *Aspergillus fumigatus*. Antimicrob Agents Chemother 2012; 56: 4870–5. 10.1128/AAC.00514-12 22751542 PMC3421857

[79] Parent-Michaud M, Dufresne PJ, Fournier E et al Prevalence and mechanisms of azole resistance in clinical isolates of *Aspergillus* section *Fumigati* species in a Canadian tertiary care centre, 2000 to 2013. J Antimicrob Chemother 2020; 75: 849–58. 10.1093/jac/dkz534 31891387 PMC7069474

[80] Negri CE, Gonçalves SS, Xafranski H et al Cryptic and rare *Aspergillus* species in Brazil: prevalence in clinical samples and in vitro susceptibility to triazoles. J Clin Microbiol 2014; 52: 3633–40. 10.1128/JCM.01582-14 25078909 PMC4187744

[81] Chen J, Li H, Li R et al Mutations in the cyp51A gene and susceptibility to itraconazole in *Aspergillus fumigatus* serially isolated from a patient with lung aspergilloma. J Antimicrob Chemother 2005; 55: 31–7. 10.1093/jac/dkh507 15563516

[82] Hsueh PR, Lau YJ, Chuang YC et al Antifungal susceptibilities of clinical isolates of Candida species, cryptococcus neoformans, and *Aspergillus* species from Taiwan: surveillance of multicenter antimicrobial resistance in Taiwan program data from 2003. Antimicrob Agents Chemother 2005; 49: 512–7. 10.1128/AAC.49.2.512-517.2005 15673726 PMC547329

[83] Meneau I, Sanglard D. Azole and fungicide resistance in clinical and environmental *Aspergillus fumigatus* isolates. Med Mycol 2005; 43: S307–11. 10.1080/13693780500090826 16110825

[84] Arabatzis M, Kambouris M, Kyprianou M et al Polyphasic identification and susceptibility to seven antifungals of 102 Aspergillus isolates recovered from immunocompromised hosts in Greece. Antimicrob Agents Chemother 2011; 55: 3025–30. 10.1128/AAC.01491-10 21444701 PMC3101447

[85] Kidd SE, Goeman E, Meis JF et al Multi-triazole-resistant *Aspergillus fumigatus* infections in Australia. Mycoses 2015; 58: 350–5. 10.1111/myc.12324 25885568

[86] Gonzalez-Lara MF, Roman-Montes CM, Diaz-Lomeli P et al Azole resistance and cyp51A mutation screening in *Aspergillus fumigatus* in Mexico. J Antimicrob Chemother 2019; 74: 2047–50. 10.1093/jac/dkz121 31220262

[87] Resendiz-Sharpe A, Dewaele K, Merckx R et al Triazole-resistance in environmental *Aspergillus fumigatus* in Latin American and African countries. J Fungi 2021; 7: 292. 10.3390/jof7040292 PMC8070258 33921497

[88] Wiederhold NP, Gil VG, Gutierrez F et al First detection of TR34 L98H and TR46 Y121F T289A Cyp51 mutations in *Aspergillus fumigatus* isolates in the United States. J Clin Microbiol 2016; 54: 168–71. 10.1128/JCM.02478-15 26491179 PMC4702720

[89] Brito Devoto T, Hermida-Alva K, Posse G et al High prevalence of triazole-resistant *Aspergillus fumigatus* sensu stricto in an Argentinean cohort of patients with cystic fibrosis. Mycoses 2020; 63: 937–41. 10.1111/myc.13139 32648614

[90] Isla G, Leonardelli F, Tiraboschi IN et al First clinical isolation of an azole-resistant *Aspergillus fumigatus* isolate harboring a TR46 Y121F T289A mutation in South America. Antimicrob Agents Chemother 2018; 62: e00872-18. 10.1128/AAC.00872-18 30082288 PMC6153829

[91] Leonardelli F, Theill L, Nardin ME et al First itraconazole resistant *Aspergillus fumigatus* clinical isolate harbouring a G54E substitution in Cyp51Ap in South America. Rev Iberoam Micol 2017; 34: 46–8. 10.1016/j.riam.2016.05.005 28087293

[92] Pontes L, Augusto C, Beraquet G et al *Aspergillus fumigatus* clinical isolates carrying CYP51A with TR34/L98H/S297T/F495I substitutions detected after four-year retrospective azole resistance screening in Brazil. Antimicrob Agents Chemother 2020; 64: e02059-19. 10.1128/AAC.02059-19 31871090 PMC7038256

[93] Denardi L, Melchers W, Zoll J et al First report of azole-resistant *Aspergillus fumigatus* harboring TR34/L98H and M220R in Brazil. 20th Congr Int Soc Human Anim Mycol (ISHAM 20th) 2018. Abstract S6.6c.

[94] Álvarez Duarte E, Cepeda N, Miranda J. Azole resistance in a clinical isolate of *Aspergillus fumigatus* from Chile. Rev Iberoam Micol 2024; 41: 7–12. 10.1016/j.riam.2024.04.003 39304433

[95] Alvarez-Moreno C, Lavergne RA, Hagen F et al Azole-resistant *Aspergillus fumigatus* harboring TR 34 /L98H, TR 46 /Y121F/T289A and TR 53 mutations related to flower fields in Colombia. Sci Rep 2017; 7: 45631. 10.1038/srep45631 28358115 PMC5372364

[96] Alvarez-Moreno C, Lavergne R-A, Hagen F et al Fungicide-driven alterations in azole-resistant *Aspergillus fumigatus* are related to vegetable crops in Colombia, South America. Mycologia 2019; 111: 217–24. 10.1080/00275514.2018.1557796 30896313

[97] Le Pape P, Lavergne RA, Morio F et al Multiple fungicide-driven alterations in azole-resistant *Aspergillus fumigatus*, Colombia, 2015. Emerg Infect Dis 2016; 22: 156–7. 10.3201/eid2201.150978 26690795 PMC4696698

[98] Bustamante B, Illescas LR, Posadas A et al Azole resistance among clinical isolates of *Aspergillus fumigatus* in Lima-Peru. Med Mycol 2020; 58: 54–60. 10.1093/mmy/myz032 31329931

[99] Huseynov R, Javadli S, Osmanov A et al TR34/L98H mutation in *Aspergillus fumigatus* isolate: first report in Azerbaijan. J Fungal Biol 2025; 15: 98–113. 10.5943/cream/15/1/7

[100] Lockhart SR, Frade JP, Etienne KA et al Azole resistance in *Aspergillus fumigatus* isolates from the ARTEMIS global surveillance study is primarily due to the TR/L98H mutation in the cyp51A gene. Antimicrob Agents Chemother 2011; 55: 4465–8. 10.1128/AAC.00185-11 21690285 PMC3165364

[101] Chen Y, Wang H, Lu Z et al Emergence of TR46/Y121F/T289A in an *Aspergillus fumigatus* isolate from a Chinese patient. Antimicrob Agents Chemother 2015; 59: 7148–50. 10.1128/AAC.00887-15 26282417 PMC4604399

[102] Xu H, Chen W, Li L et al Clinical itraconazole-resistant strains of *Aspergillus fumigatus*, isolated serially from a lung aspergilloma patient with pulmonary tuberculosis, can be detected with real-time PCR method. Mycopathologia 2010; 169: 193–9. 10.1007/s11046-009-9249-x 19888672

[103] Chowdhary A, Kathuria S, Xu J et al Clonal expansion and emergence of environmental multiple-triazole-resistant *Aspergillus fumigatus* strains carrying the TR34/L98H mutations in the cyp51A gene in India. PLoS One 2012; 7: e52871. 10.1371/journal.pone.0052871 23285210 PMC3532406

[104] Chowdhary A, Sharma C, Kathuria S et al Azole-resistant *Aspergillus fumigatus* with the environmental TR46/Y121F/T289A mutation in India. J Antimicrob Chemother 2014; 69: 555–7. 10.1093/jac/dkt397 24084639

[105] Chowdhary A, Sharma C, Kathuria S et al Prevalence and mechanism of triazole resistance in *Aspergillus fumigatus* in a referral chest hospital in Delhi, India and an update of the situation in Asia. Front Microbiol 2015; 6: 428. 10.3389/fmicb.2015.00428 26005442 PMC4424976

[106] Badali H, Vaezi A, Haghani I et al Environmental study of azole-resistant *Aspergillus fumigatus* with TR34/L98H mutations in the cyp51A gene in Iran. Mycoses 2013; 56: 659–63. 10.1111/myc.12089 23668594

[107] Seyedmousavi S, Hashemi SJ, Zibafar E et al Azole-resistant *Aspergillus fumigatus*, Iran. Emerg Infect Dis 2013; 19: 832–4. 10.3201/eid1905.130075 23697829 PMC3647519

[108] Ahangarkani F, Puts Y, Nabili M et al First azole-resistant *Aspergillus fumigatus* isolates with the environmental TR46/Y121F/T289A mutation in Iran. Mycoses 2020; 63: 430–6. 10.1111/myc.13064 32056319 PMC7217147

[109] Khojasteh S, Abastabar M, Haghani I et al Five-year surveillance study of clinical and environmental triazole-resistant *Aspergillus fumigatus* isolates in Iran. Mycoses 2023; 66: 98–105. 10.1111/myc.13535 36196507

[110] Toyotome T, Hagiwara D, Kida H et al First clinical isolation report of azole-resistant *Aspergillus fumigatus* with TR34/L98H-type mutation in Japan. J Infect Chemother 2017; 23: 579–81. 10.1016/j.jiac.2016.12.004 28109740

[111] Hagiwara D, Takahashi H, Fujimoto M et al Multi-azole resistant *Aspergillus fumigatus* harboring Cyp51A TR46/Y121F/T289A isolated in Japan. J Infect Chemother 2016; 22: 577–9. 10.1016/j.jiac.2016.01.015 26898666

[112] Lee HJ, Cho SY, Lee DG et al TR34/L98H mutation in CYP51A gene in *Aspergillus fumigatus* clinical isolates during posaconazole prophylaxis: first case in Korea. Mycopathologia 2018; 183: 731–6. 10.1007/s11046-018-0271-8 29858759 PMC6096900

[113] Ahmad S, Khan Z, Hagen F et al Occurrence of triazole-resistant *Aspergillus fumigatus* with TR34/L98H mutations in outdoor and hospital environment in Kuwait. Environ Res 2014; 133: 20–6. 10.1016/j.envres.2014.05.009 24906064

[114] Asadzadeh M, Alobaid K, Ahmad S et al First report of azole-resistant *Aspergillus fumigatus* with TR46/Y121F/T289A mutations in Kuwait and an update on their occurrence in the Middle East. J Fungi 2023; 9: 784. 10.3390/jof9080784 PMC10455753 37623555

[115] Munir G, Farhad S, Alam S et al Epidemiology and susceptibility profile of *Aspergillus* species: an experience from tertiary care hospital. Pak J Med Health Sci 2022; 16: 634–6. 10.53350/pjmhs22167634

[116] Wu CJ, Wang HC, Lee JC et al Azole-resistant *Aspergillus fumigatus* isolates carrying TR34/L98H mutations in Taiwan. Mycoses 2015; 58: 544–9. 10.1111/myc.12354 26214171

[117] Tangwattanachuleeporn M, Minarin N, Saichan S et al Prevalence of azole-resistant *Aspergillus fumigatus* in the environment of Thailand. Med Mycol 2017; 55: 429–35. 10.1093/mmy/myw090 27664994

[118] Daloh M, Wisessombat S, Pinchai N et al High prevalence and genetic diversity of a single ancestral origin azole-resistant *Aspergillus fumigatus* in indoor environments at Walailak University, Southern Thailand. Environ Microbiol 2022; 24: 4641–51. 10.1111/1462-2920.16154 36254865

[119] Duong T-MN, Le T-V, Tran K-LH et al Azole-resistant *Aspergillus fumigatus* is highly prevalent in the environment of Vietnam, with marked variability by land use type. Environ Microbiol 2021; 23: 7632–42. 10.1111/1462-2920.15660 34232541

[120] Lass-Flörl C, Mayr A, Aigner M et al A nationwide passive surveillance on fungal infections shows a low burden of azole resistance in molds and yeasts in Tyrol, Austria. Infection 2018; 46: 701–4. 10.1007/s15010-018-1170-0 29971692 PMC6182458

[121] van der Linden JWM, Arendrup MC, Warris A et al Prospective multicenter international surveillance of azole resistance in *Aspergillus fumigatus*. Emerg Infect Dis 2015; 21: 1041–4. 10.3201/eid2106.140717 25988348 PMC4451897

[122] Vermeulen E, Maertens J, Schoemans H et al Azole-resistant *Aspergillus fumigatus* due to TR46/ Y121F/T289A mutation emerging in Belgium, July 2012. Euro Surveill 2012; 17: 20326. 10.2807/ese.17.48.20326-en 23218390

[123] Holíková K . Azole Resistance in Aspergillus Fumigatus: Genetic Background and Mechanisms of Spread in the Czech Republic. Univerzita Karlova Přírodovědecká fakulta, Prague, 2023.

[124] Mortensen KL, Jensen RH, Johansen HK et al Aspergillus species and other molds in respiratory samples from patients with cystic fibrosis: a laboratory-based study with focus on *Aspergillus fumigatus* azole resistance. J Clin Microbiol 2011; 49: 2243–51. 10.1128/JCM.00213-11 21508152 PMC3122734

[125] Astvad KMT, Jensen RH, Hassan TM et al First detection of TR46/Y121F/T289A and TR34/L98H alterations in *Aspergillus fumigatus* isolates from azole-naive patients in Denmark despite negative findings in the environment. Antimicrob Agents Chemother 2014; 58: 5096–101. 10.1128/AAC.02855-14 24936595 PMC4135837

[126] Morio F, Aubin GG, Danner-Boucher I et al High prevalence of triazole resistance in *Aspergillus fumigatus*, especially mediated by TR/L98H, in a French cohort of patients with cystic fibrosis. J Antimicrob Chemother 2012; 67: 1870–3. 10.1093/jac/dks160 22581906

[127] Lavergne RA, Morio F, Danner-Boucher I et al One year prospective survey of azole resistance in *Aspergillus fumigatus* at a French cystic fibrosis reference centre: prevalence and mechanisms of resistance. J Antimicrob Chemother 2019; 74: 1884–9. 10.1093/jac/dkz144 31038164

[128] Lavergne RA, Morio F, Favennec L et al First description of azole-resistant *Aspergillus fumigatus* due to TR46/Y121F/T289A mutation in France. Antimicrob Agents Chemother 2015; 59: 4331–5. 10.1128/AAC.00127-15 25918139 PMC4468656

[129] Hamprecht A, Buchheidt D, Vehreschild JJ et al Azole-resistant invasive aspergillosis in a patient with acute myeloid leukaemia in Germany. Euro Surveill 2012; 17: 20262. 10.2807/ese.17.36.20262-en 22971327

[130] Rath PM, Buchheidt D, Spiess B et al First reported case of azole-resistant *Aspergillus fumigatus* due to the TR/L98H mutation in Germany. Antimicrob Agents Chemother 2012; 56: 6060–1. 10.1128/AAC.01017-12 22890769 PMC3486539

[131] Fischer J, van Koningsbruggen-Rietschel S, Rietschel E et al Prevalence and molecular characterization of azole resistance in *Aspergillus* spp. isolates from German cystic fibrosis patients. J Antimicrob Chemother 2014; 69: 1533–6. 10.1093/jac/dku009 24486872

[132] Steinmann J, Hamprecht A, Vehreschild MJGT et al Emergence of azole-resistant invasive aspergillosis in HSCT recipients in Germany. J Antimicrob Chemother 2014; 70: 1522–6. 10.1093/jac/dku566 25630644

[133] Siopi M, Rivero-Menendez O, Gkotsis G et al Nationwide surveillance of azole-resistant *Aspergillus fumigatus* environmental isolates in Greece: detection of pan-azole resistance associated with the TR46/Y121F/T289A cyp51A mutation. J Antimicrob Chemother 2020; 75: 3181–8. 10.1093/jac/dkaa316 32814940

[134] Dunne K, Hagen F, Pomeroy N et al Intercountry transfer of triazole-resistant *Aspergillus fumigatus* on plant bulbs. Clin Infect Dis 2017; 65: 147–9. 10.1093/cid/cix257 28369271

[135] Mohamed A, Hassan T, Trzos-Grzybowska M et al Multi-triazole-resistant *Aspergillus fumigatus* and SARS-CoV-2 co-infection: a lethal combination. Med Mycol Case Rep 2021; 31: 11–4. 10.1016/j.mmcr.2020.06.005 32837879 PMC7319628

[136] Verweij P, Mellado E, Melchers W. Multiple-triazole-resistant aspergillosis. N Engl J Med 2007; 356: 1481–3. 10.1056/NEJMc061720 17409336

[137] Buil JB, Snelders E, Denardi LB et al Trends in azole resistance in *Aspergillus fumigatus*, The Netherlands, 1994–2016. Emerg Infect Dis 2019; 25: 176–8. 10.3201/eid2501.171925 30561296 PMC6302600

[138] Van Der Linden JWM, Camps SMT, Kampinga GA et al Aspergillosis due to voriconazole highly resistant *Aspergillus fumigatus* and recovery of genetically related resistant isolates from domiciles. Clin Infect Dis 2013; 57: 513–20. 10.1093/cid/cit320 23667263

[139] Magnus E, Henriksen N. Pathogenic Fungi in Norway-Screening for Azole-Resistant Aspergillus Fumigatus in Norwegian Barns. Norwegian University of Life Sciences, 2021.

[140] Brillowska-Dąbrowska A, Mroczyńska M, Nawrot U et al Examination of cyp51A and cyp51B expression level of the first Polish azole resistant clinical *Aspergillus fumigatus* isolate. Acta Biochim Pol 2015; 62: 837–9. 10.18388/abp.2015_1143 26636140

[141] Pinto E, Monteiro C, Maia M et al *Aspergillus* species and antifungals susceptibility in clinical setting in the north of Portugal: cryptic species and emerging azoles resistance in *A. fumigatus*. Front Microbiol 2018; 9: 1656. 10.3389/fmicb.2018.01656 30083151 PMC6065200

[142] Sharma C, Hagen F, Moroti R et al Triazole-resistant *Aspergillus fumigatus* harbouring G54 mutation: is it de novo or environmentally acquired? J Glob Antimicrob Resist 2015; 3: 69–74. 10.1016/j.jgar.2015.01.005 27873672

[143] Klyasova G, Malchikova A, Khrulnova S. The first case of *Aspergillus fumigatus* with high-level resistance to voriconazole due to the TR46/Y121F/T289A mutation in the Russian Federation. Diagn Microbiol Infect Dis 2025; 111: 116624. 10.1016/j.diagmicrobio.2024.116624 39644541

[144] Mellado E, De La Camara R, Buendía B et al Breakthrough pulmonary *Aspergillus fumigatus* infection with multiple triazole resistance in a Spanish patient with chronic myeloid leukemia. Rev Iberoam Micol 2013; 30: 64–8. 10.1016/j.riam.2012.09.002 22986228

[145] Pelaez T, Monteiro MC, Garcia-Rubio R et al First detection of *Aspergillus fumigatus* azole-resistant strain due to Cyp51A TR46/Y121F/T289A in an azole-naive patient in Spain. New Microbes New Infect 2015; 6: 33–4. 10.1016/j.nmni.2015.04.005 26082842 PMC4459865

[146] Escribano P, Recio S, Peláez T et al *Aspergillus fumigatus* strains with mutations in the cyp51A gene do not always show phenotypic resistance to itraconazole, voriconazole, or posaconazole. Antimicrob Agents Chemother 2011; 55: 2460–2. 10.1128/AAC.01358-10 21321141 PMC3088257

[147] Riat A, Plojoux J, Gindro K et al Azole resistance of environmental and clinical *Aspergillus fumigatus* isolates from Switzerland. Antimicrob Agents Chemother 2018; 62: e02088-17. 10.1128/AAC.02088-17 29437612 PMC5913999

[148] Schürch S, Gindro K, Schnee S et al Occurrence of *Aspergillus fumigatus* azole resistance in soils from Switzerland. Med Mycol 2023; 61: myad110. 10.1093/mmy/myad110 37930839 PMC10653585

[149] Moore CB, Novak-Frazer L, Muldoon E et al First isolation of the pan-azole-resistant *Aspergillus fumigatus* cyp51A TR46/Y121F/T289A mutant in a UK patient. Int J Antimicrob Agents 2017; 49: 512–4. 10.1016/j.ijantimicag.2017.01.004 28193463

[150] Howard SJ, Webster I, Moore CB et al Multi-azole resistance in *Aspergillus fumigatus*. Int J Antimicrob Agents 2006; 28: 450–3. 10.1016/j.ijantimicag.2006.08.017 17034993

[151] Yerbanga IW, Lagrou K, Merckx R et al First detection of triazole-resistant *Aspergillus fumigatus* harbouring the TR34/L98H *Cyp51A* mutation in Burkina Faso. Mycoses 2024; 67: e13732. 10.1111/myc.13732 38712846

[152] Yerbanga IW, Resendiz-Sharpe A, Bamba S et al First investigative study of azole-resistant *Aspergillus fumigatus* in the environment in Burkina Faso. Int J Environ Res Public Health 2021; 18: 2250. 10.3390/ijerph18052250 33668719 PMC7956412

[153] Kemoi EK, Nyerere A, Gross U et al Diversity of azoles resistant *Aspergillus* species isolated from experience and naïve soils in Nairobi county and Naivasha sub-county Kenya. Eur Sci J 2017; 13: 301. 10.19044/esj.2017.v13n36p301

[154] Kemoi EK, Nyerere A, Bii CC. Triazole-resistant aspergillus fumigatus from fungicide-experienced soils in Naivasha subcounty and Nairobi county, Kenya. Int J Microbiol 2018; 2018: 7147938. 10.1155/2018/7147938 30046310 PMC6038473

[155] Moglad E, Saeed S, Saeed H et al Molecular characterization and antifungal susceptibility of *Aspergillus* spp. among patients with underlying lung diseases. Trop Med Infect Dis 2022; 7: 274. 10.3390/tropicalmed7100274 36288015 PMC9612272

[156] Zhou S, Ismail MAI, Buil JB et al Fungi involved in rhinosinusitis in arid regions: insights from molecular identification and antifungal susceptibility. Microbiol Spectr 2023; 11: e01831-23. 10.1128/spectrum.01831-23 37772821 PMC10580872

[157] Chowdhary A, Sharma C, van den Boom M et al Multi-azole-resistant *Aspergillus fumigatus* in the environment in Tanzania. J Antimicrob Chemother 2014; 69: 2979–83. 10.1093/jac/dku259 25006238

[158] Mushi MF, Buname G, Bader O et al *Aspergillus fumigatus* carrying TR34/L98H resistance allele causing complicated suppurative otitis media in Tanzania: call for improved diagnosis of fungi in sub-Saharan Africa. BMC Infect Dis 2016; 16: 464. 10.1186/s12879-016-1796-4 27589956 PMC5009654

[159] Morris AJ, McKinney WP, Roberts SA et al Appearance of environment-linked azole resistance in the *Aspergillus fumigatus* complex in New Zealand. Mycoses 2025; 68: e70104. 10.1111/myc.70104 40857214 PMC12379841

[160] McKinney WP, Vesty A, Sood J et al The emergence of azole resistance in *Aspergillus fumigatus* complex in New Zealand. NZ Med J 2021; 134: 41–51. https://nzmj.org.nz/media/pages/journal/vol-134-no-1536/the-emergence-of-azole-resistance-in-aspergillus-fumigatus-complex-in-new-zealand/74ef7271b8-1696473312/the-emergence-of-azole-resistance-in-aspergillus-fumigatus-complex-in-new-zealand.pdf. 34140712

[161] Hare RK, Gertsen JB, Astvad KMT et al In vivo selection of a unique tandem repeat mediated azole resistance mechanism (TR120) in *Aspergillus fumigatus* cyp51A, Denmark. Emerg Infect Dis 2019; 25: 577–80. 10.3201/eid2503.180297 30789127 PMC6390761

[162] Shelton JMG, Collins R, Uzzell CB et al Citizen science surveillance of triazole-resistant *Aspergillus fumigatus* in United Kingdom residential garden soils. Appl Environ Microbiol 2022; 88: e02061-21. 10.1128/aem.02061-21 34986003 PMC8862786

[163] Hagiwara D . Isolation of azole-resistant *Aspergillus fumigatus* from imported plant bulbs in Japan and the effect of fungicide treatment. J Pestic Sci 2020; 45: 147–50. 10.1584/jpestics.D20-017 32913417 PMC7453303

[164] Uehara S, Takahashi H, Nishino Y et al Genetic analysis of azole-resistant *Aspergillus fumigatus* isolated from domestic and imported tulip bulbs in Japan. J Glob Antimicrob Resist 2025; 44: 306–13. 10.1016/j.jgar.2025.07.006 40651719

[165] Bueid A, Howard SJ, Moore CB et al Azole antifungal resistance in *Aspergillus fumigatus*: 2008 and 2009. J Antimicrob Chemother 2010; 65: 2116–8. 10.1093/jac/dkq279 20729241

[166] Bongomin F, Harris C, Hayes G et al Twelve-month clinical outcomes of 206 patients with chronic pulmonary aspergillosis. PLoS One 2018; 13: e0193732. 10.1371/journal.pone.0193732 29634721 PMC5892866

[167] Verweij PE, Lestrade PPA, Melchers WJG et al Azole resistance surveillance in Aspergillus fumigatus: beneficial or biased? J Antimicrob Chemother 2016; 71: 2079–82. 10.1093/jac/dkw259 27494831

[168] Lucio J, Alcazar-Fuoli L, Gil H et al Distribution of *Aspergillus* species and prevalence of azole resistance in clinical and environmental samples from a Spanish hospital during a three-year study period. Mycoses 2024; 67: e13719. 10.1111/myc.13719 38551063

[169] Korfanty G, Kazerouni A, Dixon M et al What in earth? Analyses of Canadian soil populations of *Aspergillus fumigatus*. Can J Microbiol 2024; 71: 1–13. 10.1139/cjm-2024-0083 39405583

[170] Etienne KA, Berkow EL, Gade L et al Genomic diversity of azole-resistant *Aspergillus fumigatus* in the United States. mBio 2021; 12: e01803-21. 10.1128/mBio.01803-21 34372699 PMC8406307

[171] Yang X, Chen W, Liang T et al A 20-year antifungal susceptibility surveillance (from 1999 to 2019) for *Aspergillus* spp. and proposed epidemiological cutoff values for *Aspergillus fumigatus* and *Aspergillus flavus*: a study in a tertiary hospital in China. Front Microbiol 2021; 12: 680884. 10.3389/fmicb.2021.680884 34367087 PMC8339419

[172] Singh A, Sharma B, Mahto KK et al High-frequency direct detection of triazole resistance in *Aspergillus fumigatus* from patients with chronic pulmonary fungal diseases in India. J Fungi 2020; 6: 67. 10.3390/jof6020067 PMC7345705 32443672

[173] Takazono T, Ito Y, Tashiro M et al Transition of triazole-resistant *Aspergillus fumigatus* isolates in a Japanese tertiary hospital and subsequent genetic analysis. J Infect Chemother 2021; 27: 537–9. 10.1016/j.jiac.2020.11.027 33309631

[174] Cho SY, Lee DG, Kim WB et al Epidemiology and antifungal susceptibility profile of *Aspergillus* species: comparison between environmental and clinical isolates from patients with hematologic malignancies. J Clin Microbiol 2019; 57: e02023-18. 10.1128/JCM.02023-18 31018982 PMC6595445

[175] Wang HC, Hsieh MI, Choi PC et al Species distribution and antifungal susceptibility of clinical Aspergillus isolates: a multicentre study in Taiwan, 2016–2020. Mycoses 2023; 66: 711–22. 10.1111/myc.13593 37186489

[176] Debergh H, Castelain P, Goens K et al Detection of pan-azole resistant *Aspergillus fumigatus* in horticulture and a composting facility in Belgium. Med Mycol 2024; 62: myae055. 10.1093/mmy/myae055 38769604 PMC11223581

[177] Arendrup MC, Hare RK, Jørgensen KM et al Environmental hot spots and resistance-associated application practices for azole-resistant *Aspergillus fumigatus*, Denmark, 2020–2023. Emerg Infect Dis 2024; 30: 1531–41. 10.3201/eid3008.240096 38935978 PMC11286046

[178] Simon L, Déméautis T, Dupont D et al Azole resistance in *Aspergillus fumigatus* isolates from respiratory specimens in Lyon University Hospitals, France: prevalence and mechanisms involved. Int J Antimicrob Agents 2021; 58: 106447. 10.1016/j.ijantimicag.2021.106447 34619334

[179] Seufert R, Sedlacek L, Kahl B et al Prevalence and characterization of azole-resistant *Aspergillus fumigatus* in patients with cystic fibrosis: a prospective multicentre study in Germany. J Antimicrob Chemother 2018; 73: 2047–53. 10.1093/jac/dky147 29684150

[180] van Dijk MAM, Buil JB, Tehupeiory-Kooreman M et al Azole resistance in veterinary clinical *Aspergillus fumigatus* isolates in The Netherlands. Mycopathologia 2024; 189: 50. 10.1007/s11046-024-00850-5 38864903 PMC11169034

[181] Nawrot U, Kurzyk E, Arendrup MC et al Detection of Polish clinical *Aspergillus fumigatus* isolates resistant to triazoles. Med Mycol 2018; 56: 121–4. 10.1093/mmy/myx012 28340159

[182] Gonçalves P, Melo A, Dias M et al Azole-resistant *Aspergillus fumigatus* harboring the TR34/l98H mutation: first report in Portugal in environmental samples. Microorganisms 2021; 9: 57. 10.3390/microorganisms9010057 PMC7823791 33379247

[183] Ener B, Ergin Ç, Gülmez D et al Frequency of azole resistance in clinical and environmental strains of *Aspergillus fumigatus* in Turkey: a multicentre study. J Antimicrob Chemother 2022; 77: 1894–8. 10.1093/jac/dkac125 35445259

[184] Shelton JMG, Rhodes J, Uzzell CB et al Citizen science reveals landscape-scale exposures to multiazole-resistant *Aspergillus fumigatus* bioaerosols. Sci Adv 2023; 9: eadh8839. 10.1126/sciadv.adh8839 37478175 PMC10361594

[185] Kemoi EK, Nyerere A, Mashedi O et al Azole resistant *Aspergillus fumigatus* with TR34/L98H mutation from clinical isolates in Kenya. East Afr Med J 2023; 100: 5832–7. https://www.ajol.info/index.php/eamj/article/view/248662.

[186] Talbot JJ, Subedi S, Halliday CL et al Surveillance for azole resistance in clinical and environmental isolates of *Aspergillus fumigatus* in Australia and cyp51A homology modelling of azole-resistant isolates. J Antimicrob Chemother 2018; 73: 2347–51. 10.1093/jac/dky187 29846581

[187] Mellado E, Alcazar-Fuoli L, García-Effrón G et al New resistance mechanisms to azole drugs in *Aspergillus fumigatus* and emergence of antifungal drugs-resistant *A. fumigatus* atypical strains. Med Mycol 2006; 44: 367–71. 10.1080/13693780600902243 30408931

[188] Swain S, Ajayababu A, Chowdhary S et al Epidemiology of triazole resistant *Aspergillus fumigatus* in Asia: a systematic review and meta-analysis. Mycoses 2025; 68: e70099. 10.1111/myc.70099 40792442

[189] Berkow EL, Nunnally NS, Bandea A et al Detection of TR 34 /L98H CYP51A mutation through passive surveillance for azole-resistant *Aspergillus fumigatus* in the United States from 2015 to 2017. Antimicrob Agents Chemother 2018; 62: e02240-17. 10.1128/AAC.02240-17 29463545 PMC5923109

[190] Pham CD, Reiss E, Hagen F et al Passive surveillance for azole-resistant *Aspergillus fumigatus*, United States, 2011–2013. Emerg Infect Dis 2014; 20: 1498–503. 10.3201/eid2009.140142 25148217 PMC4178384

[191] Hurst SF, Berkow EL, Stevenson KL et al Isolation of azole-resistant *Aspergillus fumigatus* from the environment in the south-eastern USA. J Antimicrob Chemother 2017; 72: 2443–6. 10.1093/jac/dkx168 28575384 PMC11935745

[192] Lahr J, Buij R, Katagira F et al Pesticides in the Southern Agricultural Growth Corridor of Tanzania (SAGCOT): A Scoping Study of Current and Future Use, Associated Risks and Identification of Actions for Risk Mitigation. Alterra-rapport - Wageningen University and Research Centre 2016; 2760: 71. https://research.wur.nl/en/publications/07740271-eb3a-4f65-aa98-e95e0bb2aed8.

